# Prediction of major adverse cardiovascular events in patients with acute coronary syndrome: Development and validation of a non-invasive nomogram model based on autonomic nervous system assessment

**DOI:** 10.3389/fcvm.2022.1053470

**Published:** 2022-11-03

**Authors:** Jun Wang, Xiaolin Wu, Ji Sun, Tianyou Xu, Tongjian Zhu, Fu Yu, Shoupeng Duan, Qiang Deng, Zhihao Liu, Fuding Guo, Xujun Li, Yijun Wang, Lingpeng Song, Hui Feng, Xiaoya Zhou, Hong Jiang

**Affiliations:** ^1^Department of Cardiology, Renmin Hospital of Wuhan University, Wuhan, China; ^2^Cardiac Autonomic Nervous System Research Center of Wuhan University, Wuhan, China; ^3^Hubei Key Laboratory of Autonomic Nervous System Modulation, Wuhan, China; ^4^TaiKang Center for Life and Medical Sciences, Wuhan University, Wuhan, China; ^5^Cardiovascular Research Institute, Wuhan University, Wuhan, China; ^6^Hubei Key Laboratory of Cardiology, Wuhan, China; ^7^Information Center, Renmin Hospital of Wuhan University, Wuhan, China; ^8^Department of Cardiology, Institute of Cardiovascular Diseases, Xiangyang Central Hospital, Affiliated Hospital of Hubei University of Arts and Science, Xiangyang, Hubei, China

**Keywords:** acute coronary syndrome, major adverse cardiovascular events, nomogram, prediction nomogram, outcomes, autonomic nervous system

## Abstract

**Background:**

Disruption of the autonomic nervous system (ANS) can lead to acute coronary syndrome (ACS). We developed a nomogram model using heart rate variability (HRV) and other data to predict major adverse cardiovascular events (MACEs) following emergency coronary angiography in patients with ACS.

**Methods:**

ACS patients admitted from January 2018 to June 2020 were examined. Holter monitors were used to collect HRV data for 24 h. Coronary angiograms, clinical data, and MACEs were recorded. A nomogram was developed using the results of Cox regression analysis.

**Results:**

There were 439 patients in a development cohort and 241 in a validation cohort, and the mean follow-up time was 22.80 months. The nomogram considered low-frequency/high-frequency ratio, age, diabetes, previous myocardial infarction, and current smoking. The area-under-the-curve (AUC) values for 1-year MACE-free survival were 0.790 (95% CI: 0.702–0.877) in the development cohort and 0.894 (95% CI: 0.820–0.967) in the external validation cohort. The AUCs for 2-year MACE-free survival were 0.802 (95% CI: 0.739–0.866) in the development cohort and 0.798 (95% CI: 0.693–0.902) in the external validation cohort. Development and validation were adequately calibrated and their predictions correlated with the observed outcome. Decision curve analysis (DCA) showed the model had good discriminative ability in predicting MACEs.

**Conclusion:**

Our validated nomogram was based on non-invasive ANS assessment and traditional risk factors, and indicated reliable prediction of MACEs in patients with ACS. This approach has potential for use as a method for non-invasive monitoring of health that enables provision of individualized treatment strategies.

## Introduction

Coronary artery disease (CAD) is the major cause of death worldwide (1, 2). Even though risk-factor targeted management and revascularization have dramatically improved the outcomes for these patients, especially those with acute coronary syndrome (ACS), these patients remain at high risk for morbidity and mortality (3–5). Strategies for risk stratification and risk-adjusted management of ACS have been a major focus of researchers during the past decade (6, 7). Therefore, timely diagnosis, early treatment, and early detection of risk factors have great clinical significance in cardiovascular care (6, 7). Notably, remote monitoring of cardiovascular health has received a great deal of attention for management of patients with cardiovascular disease (8).

During pathological conditions, there is evidence that disruption of the autonomic nervous system (ANS) potentially affects the growth of atherosclerotic plaques, and these findings have significant prognostic implications for patients with ACS (9–11). In clinical settings, non-invasive and ambulatory monitoring techniques that measure heart rate variability (HRV) provide insight into autonomic modulation of cardiac function (12). Moreover, due to its value as an independent prognostic indicator, measurements of HRV have potential use in clinical evaluations and risk stratification of high risk patients (12). Novel and effective digital monitoring risk-assessment tools that are used to monitor general health status should be simple, easy-to-learn, and easy for patients to operate.

A previous report showed that risk stratification using an objective and non-invasive electrocardiogram and a HRV-based tool for 1 month reduced adverse clinical outcomes and improved myocardial infarct score more than thrombolysis, although this study did not perform validation using an external cohort (13). In addition, there are several well-established models for determining the risk of cardiovascular diseases in United States and Europe, but these have generally not assessed the ANS (14). Moreover, the development of risk prediction models and recommendations regarding risk assessment in clinical guidelines has rarely been reviewed from a global perspective.

Therefore, the current study used a systematic and integrated approach that incorporated HRV data with classical risk factors to develop a prognostic model that accurately and non-invasively predicts outcomes in patients with ACS who received emergency coronary angiography.

## Materials and methods

### Patient population

**FIGURE 1:**
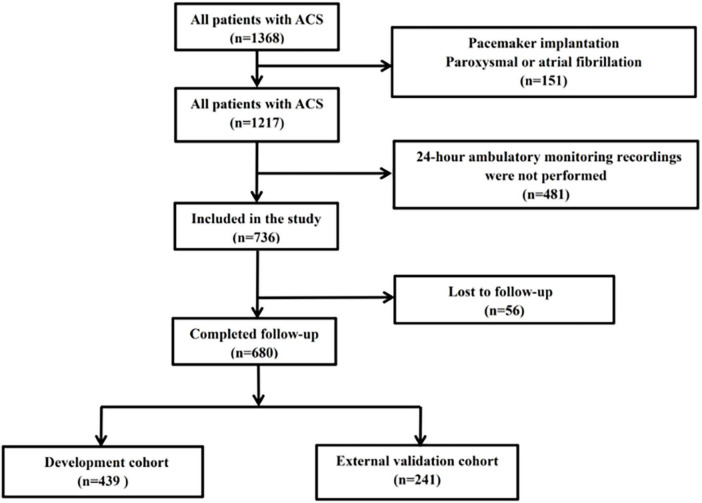
Disposition of patients with ACS (*n* = 1,368), and establishment of the development cohort (*n* = 439) and external validation cohort (*n* = 241).

The derivation set consisted of 439 consecutive patients who had ACS and received emergency coronary angiography and successful Holter monitoring for 24 h at Renmin Hospital of Wuhan University from January 2018 to June 2020. The validation set consisted of 241 consecutive patients who had ACS and received emergency coronary angiography and successful Holter monitoring for 24 h at Xiangyang Central Hospital from January 2018 to June 2020 (Figure 1). International guidelines were used to diagnose ACS (15). Patients with the following conditions were excluded: chronic coronary syndromes, delay in coronary angiography due to valvular heart disease, congenital heart disease, pacemaker implantation, paroxysmal or atrial fibrillation, chronic or acute phases of inflammation, malignant tumor, or lack of 24-h ambulatory HRV monitoring. Ethical approval was granted for the study by the local ethics committee (Renmin Hospital of Wuhan University; No. WDRY2022-K075; Xiangyang Central Hospital; No.2022-066), which waived the need for patient consent.

### Biochemical tests

Prior to emergency coronary angiography, blood biomarkers of inflammation [neutrophils, lymphocytes, neutrophil to lymphocyte ratio (NLR), and platelet-to-lymphocyte ratio (PLR)], kidney function [serum creatinine, uric acid, and estimated glomerular filtration rate (eGFR)] and glucose were measured. Blood was collected in the early morning after fasting to determine the lipid profile {high-density lipoprotein cholesterol [HDL-C], low-density lipoprotein cholesterol [LDL-C], total cholesterol [TC], triglyceride [TG], and lipoprotein a [Lp (a)]}.

### Holter monitoring

As previously described, each patient underwent Holter monitoring for 24 h after angiography to evaluate the ANS by analysis of 24-h mean heart rate and variables of HRV (16–18). An average 5-min short-term HRV analysis was conducted. R peak detection was used to identify normal sinus RR intervals (SDNN), and the standard deviation of all normal SDNN, root mean square successive difference (RMSSD), and standard deviation average of NN intervals (SDANN) were then calculated. The percentage of the time that the difference between adjacent normal RR intervals was greater than 50 ms over the total number of NN intervals (PNN50) was also calculated. For power spectrum analysis of HRV, the high-frequency (HF) spectrum ranged from 0.15 to 0.4 Hz; the low-frequency (LF) spectrum ranged from 0.04 to 0.15 Hz; and the very low-frequency (VLF) spectrum ranged from 0.003 to 0.04 Hz. Normalized units of the LF band (nLF) were calculated as: 100 × LF/(total power − VLF); normalized units of the HF band (HFn) were calculated as: 100 × HF/(total power − VLF); the ratio of the two values was calculated as: LF/HF.

### Coronary angiography

Coronary angiography was performed immediately after admission for ACS by experienced senior cardiologists who used standard treatment protocols. In accordance with guidelines, loading doses of aspirin, ticagrelor, or clopidogrel were given to patients with ACS and before operation (15). The percutaneous coronary intervention (PCI) procedures were performed by a team of highly experienced senior physicians in accordance with each patient’s coronary anatomy and clinical condition. After the procedure, each patient received standard treatment regimens based on guideline recommendations, including modern antiplatelet therapy and standard-intensity statins, and received regular follow-up at the clinic after discharge.

### Follow-up

Follow-up, which consisted of telephone calls and/or clinic visitations, was used to determine when an endpoint occurred. The primary endpoint was a major adverse cardiac events (MACEs), defined as the composite of cardiac mortality, recurrence of myocardial infarction, or need for revascularization. Cardiac mortality refers to death caused by any cardiac condition. Myocardial infarction refers to a new myocardial infarction in targeted or non-targeted vessels. Revascularization refers to targeted or non-targeted vessels.

### Statistical analysis

Statistical analysis was conducted using SPSS version 23 (IBM, Chicago, IL) and R software version 3.6.1 (R Foundation for Statistical Computing, Vienna). Means and standard deviations (SDs) were presented for continuous variables with normal distributions, and medians and interquartile ranges (IQRs) for continuous variables with skewed distributions. For analysis of continuous variables with normal distributions, the *t*-test was used; for analysis of continuous variables with non-normal distributions, the Mann-Whitney *U*-test or the Kruskal-Wallis test was used. Analysis of categorical variables was performed using the χ^2^-test. In all comparisons, a *P*-value below 0.05 indicated statistical significance.

Univariate and multivariate analyses were used to identify factors independently associated with outcome (MACEs). Analysis of the statistical significance of each individual variable was performed using univariate analysis. This was followed by multivariate analysis of variables that were significant in the univariate analysis to identify significant and independent predictors of MACEs.

Predictive nomograms were constructed using predictors that were statistically significant. R software was used to construct the nomogram using the Regression Modeling Strategies (rms) package. Risk nomograms were analyzed using receiver characteristic curves (ROC) to assess their ability to discriminate MACEs based on calculation of area under the ROC curve (19). Using the rms package, calibration curves were plotted and calculated to evaluate the calibration of the MACEs after construction of the ACS risk nomograms. Bootstrapping was performed by repeated random sampling (500 times) for internal validation of model accuracy. Decision curve analysis (DCA) was used to quantify the clinical efficacy of the nomograms based on their net benefit for different threshold probabilities (19).

## Results

### Patient characteristics

We retrospectively screened 680 patients who had ACS and received emergency angiography and divided them into a development cohort (*n* = 439) and an independent external validation cohort (*n* = 241). Among all 680 patients, the mean age was 62.9 (± 12.6) years-old and 423 patients (62.2%) were males. The two cohorts were well balanced in baseline characteristics and outcomes except for clinical presentation, LDL-C, in-hospital PCI, several results from the coronary angiography, and HRV (Table 1). The overall incidence of MACEs was 9.10% during the median follow-up time of 22.80 months, with similar incidences in the two cohorts.

**TABLE 1:** Baseline characteristics and MACEs (clinical outcome) of patients in the development and validation cohorts*.

Characteristic or outcome	Development cohort (*n* = 439)	Validation cohort (*n* = 241)	*T*, Z, or χ^2^	*P*
Male, *n* (%)	270 (61.5)	153 (63.5)	0.260	0.610
Age, years	63.32 ± 10.56	62.33 ± 11.08	1.148	0.251
Hypertension, *n* (%)	277 (63.1)	141 (58.5)	1.385	0.239
Duration of Hypertension, years	10.00 (5.00, 20.00)	10.00 (5.00, 23.00)	0.958	0.338
Diabetes, *n* (%)	105 (24.0)	47 (19.5)	1.757	0.185
Duration of diabetes, years	7.00 (3.00,10.00)	10.00 (5.00,14.00)	1.685	0.092
Current smoker, *n* (%)	148 (33.7)	94 (39.0)	1.900	0.168
Current drinker, *n* (%)	84 (19.1)	59 (24.5)	2.678	0.102
Family history of hypertension, *n* (%)	34 (7.7)	14 (5.8)	0.889	0.346
Family history of CAD, *n* (%)	28 (6.4)	15 (6.2)	0.006	0.937
Family history of diabetes, *n* (%)	34 (7.7)	19 (7.9)	0.004	0.948
Previous PCI, *n* (%)	128 (29.2)	74 (30.7)	0.179	0.673
Previous myocardial infarction, *n* (%)	49 (11.2)	27 (11.2)	0.000	0.987
Neutrophils (× 10^9^/L)	3.98 (2.98, 5.46)	4.55 (2.80, 6.40)	1.561	0.119
Lymphocytes (× 10^9^/L)	1.64 (1.27,2.16)	1.58 (1.20, 2.27)	0.290	0.772
NLR	2.33 (1.72, 3.57)	2.60 (1.42, 4.24)	0.216	0.829
PLTs (× 10^9^/L)	208.25 ± 62.20	207.51 ± 54.33	0.154	0.878
PLR	125.52 (92.27, 160.00)	122.50 (85.27, 181.58)	0.111	0.912
eGFR, mL/min/1.73 m^2^	87.56 ± 20.30	84.95 ± 18.43	1.659	0.098
Creatinine, μmol/L	72.54 ± 22.41	75.93 ± 23.11	1.865	0.063
Uric acid, mmol/L	365.78 ± 114.15	350.97 ± 99.04	1.694	0.091
Glucose, mmol/L	6.48 ± 2.85	6.64 ± 2.59	0.719	0.473
TG, mmol/L	1.44 (1.03, 2.06)	1.35 (0.96, 2.02)	1.582	0.114
TC, mmol/L	4.22 ± 1.12	4.33 ± 1.02	1.298	0.195
HDL-C, mmol/L	1.09 ± 0.29	1.10 ± 0.28	0.190	0.850
LDL-C, mmol/L	2.43 ± 0.98	2.67 ± 0.83	3.406	**0.001**
Lp (a), g/L	188.00 (78.00, 323.00)	196.45 (135.66, 269.91)	0.663	0.507
Average heart rate, bpm	68.96 ± 9.61	67.98 ± 9.30	1.278	0.202
SDNN, ms	115.21 ± 33.01	114.30 ± 29.25	0.358	0.720
SDANN, ms	77.69 ± 35.68	80.30 ± 29.32	1.026	0.305
rMSSD, ms	31.00 (22.00, 47.00)	23.00 (16.50, 31.50)	7.229	**<0.001**
Pnn50	5.37 (2.00, 9.00)	4.00 (2.00, 6.00)	3.621	**<0.001**
Normalized LF	59.65 ± 15.01	58.66 ± 22.45	0.616	0.539
Normalized HF	40.31 ± 15.01	41.38 ± 22.50	0.659	0.510
LF/HF	1.52 (0.98, 2.36)	3.16 (2.00, 4.66)	12.381	**<0.001**
Clinical Systolic Blood Pressure, mmHg	134.68 ± 19.83	133.04 ± 23.59	0.916	0.360
Clinical Diastolic Blood Pressure, mmHg	77.06 ± 12.01	78.33 ± 12.16	1.316	0.189
Heart rate (bpm)	76.88 ± 13.38	75.98 ± 16.84	0.762	0.446
MACEs, *n* (%)	37 (8.4)	24 (10.0)	0.446	0.504
Cardiac death, *n* (%)	9 (2.1)	7 (2.9)	0.494	0.482
Revascularization, *n* (%)	23 (5.2)	9 (3.7)	0.786	0.375
Myocardial infarction, *n* (%)	13 (3.0)	9 (3.7)	0.297	0.586

*Values are given as *n* (%), mean ± SD, or median (IQR).

CAD, coronary artery disease; PCI, percutaneous coronary intervention; NLR, neutrophil to lymphocyte ratio; PLT, platelet counts; PLR, platelet-to-lymphocyte ratio; eGFR, estimated glomerular filtration rate; HDL-C, high-density lipoprotein cholesterol; LDL-C, low-density lipoprotein cholesterol; TC, total cholesterol; TG, triglyceride; Lp (a), lipoprotein a; SDNN, standard deviation of all normal sinus RR intervals; SDANN, standard deviation average of normal-tonormal (NN) intervals; pNN50, percentage of the number of times that the difference between adjacent normal RR intervals > 50 ms in the total number of NN intervals; rMSSD, root mean square successive difference; LF, low-frequency; HF, high-frequency; MACEs, major adverse cardiovascular events. Bold values represent the *p* < 0.05 statistical significance.

### Potential predictors of major adverse cardiovascular events

**FIGURE 2:**
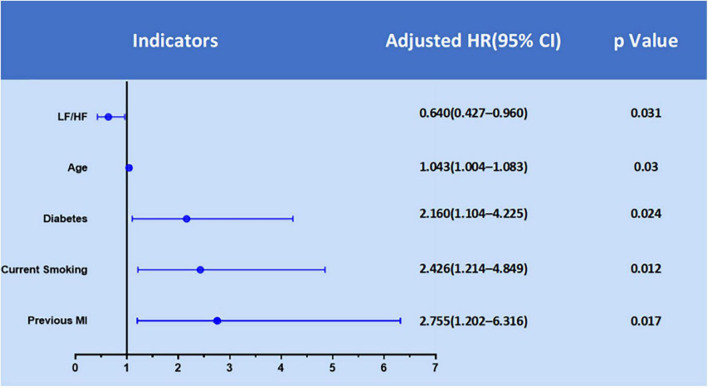
Significant predictors of MACEs in the development cohort from multivariate Cox regression analysis.

We used multivariable stepwise Cox regression model to identify significant and independent predictors of MACEs in the development cohort (Table 2). There were five significant and independent predictors of MACEs: age (HR: 1.043, 95% CI: 1.004–1.083, *P* = 0.030), diabetes (HR: 2.160, 95% CI: 1.104–4.225, *P* = 0.024), current smoker (HR: 2.426, 95% CI: 1.214–4.849, *P* = 0.012), previous myocardial infarction (HR: 2.755, 95% CI: 1.202–6.316, *P* = 0.017), and LF/HF ratio (HR: 0.640, 95% CI: 0.427–0.960, *P* = 0.031) (Figure 2).

**TABLE 2:** Univariate and multivariable Cox regression analysis of factors associated with MACEs in the development cohort.

	Univariate			Multivariate		
		
Variable	HR	95% CI	P	HR	95% CI	P
Male	0.768	0.386–1.529	0.452			
Age	1.054	1.019–1.090	**0.002**	1.043	1.004–1.083	**0.030**
Hypertension	1.409	0.696–2.851	0.341			
Diabetes	2.508	1.309–4.806	**0.006**	2.160	1.104–4.225	**0.024**
Current smoker	2.117	1.111–4.034	**0.023**	2.426	1.214–4.849	**0.012**
Current drinker	0.979	0.430–2.228	0.959			
Family history of hypertension	0.659	0.159–2.740	0.566			
Family history of CAD	0.850	0.204–3.534	0.823			
Family history of diabetes mellitus	1.018	0.313–3.314	0.977			
Previous PCI	0.968	0.301–3.112	0.956			
Previous myocardial infarction	3.684	1.820–7.457	**<0.001**	2.755	1.202–6.316	**0.017**
Neutrophils	1.024	1.004–1.046	**0.020**	1.003	0.977–1.029	0.835
Lymphocytes	1.034	0.987–1.083	0.156			
NLR	0.947	0.814–1.101	0.477			
PLT	0.995	0.990–1.001	0.079			
PLR	0.993	0.987–0.999	**0.019**	0.995	0.988–1.001	0.104
eGFR	0.984	0.971–0.996	**0.012**	0.999	0.983–1.016	0.928
Creatinine	1.011	0.999–1.022	0.062			
Uric acid	0.999	0.996–1.002	0.533			
Glucose	1.007	0.904–1.123	0.894			
TG	0.944	0.725–1.229	0.668			
TC	1.120	0.852–1.472	0.418			
HDL-C	0.793	0.254–2.479	0.690			
LDL-C	1.145	0.839–1.561	0.393			
Lp (a)	1.001	1.001–1.003	**0.005**	1.001	0.999–1.002	0.096
Average heart rate	0.999	0.966–1.034	0.970			
SDNN	0.995	0.985–1.006	0.371			
SDANN	1.001	0.991–1.009	0.986			
rMSSD	0.997	0.987–1.008	0.635			
Pnn50	0.997	0.963–1.032	0.851			
Normalized LF	0.962	0.942–0.982	<0.001			
Normalized HF	1.040	1.019–1.061	<0.001			
Normalized LF/HF	0.559	0.370–0.844	**0.006**	0.640	0.427–0.960	**0.031**
Clinical systolic blood pressure	1.015	1.001–1.031	**0.049**	1.018	0.999–1.037	0.053
Clinical diastolic blood pressure	1.004	0.977–1.031	0.777			
Average heart rate	1.003	0.980–1.027	0.792			

See Table 1. Bold values represent the *p* < 0.05 statistical significance.

### Construction of the nomogram

**FIGURE 3:**
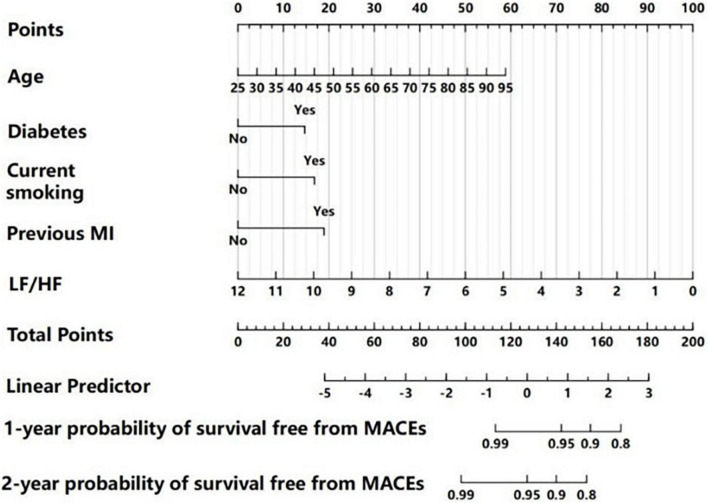
Nomogram for predicting 1 and 2-year MACE-free survival, based on multivariate Cox regression analysis. For each patient, each clinical characteristic was assigned points by drawing a vertical line from its value to the top row. The points for all five characteristics were added to determine total points middle row. Then, the total points were used to determine the 1 and 2-year probabilities of MACE-free survival by drawing a vertical line to the bottom two rows.

We then used a nomogram model to present the results of the multivariate Cox regression in predicting the 1 and 2-year probability of MACE-free survival (Figure 3). To calculate risk for a patient using this nomogram model, a vertical line is first drawn from the value of each of the five parameters to the “Points” line on the top to determine its number of points. Then, the total number of points from all five parameters is calculated (theoretical range: 0–200). Finally, the 1 and 2-year probabilities of MACE-free survival were calculated by drawing a vertical line from the “Total Points” line to the scales on the bottom two rows.

### Diagnostic performance of the nomogram

**FIGURE 4:**
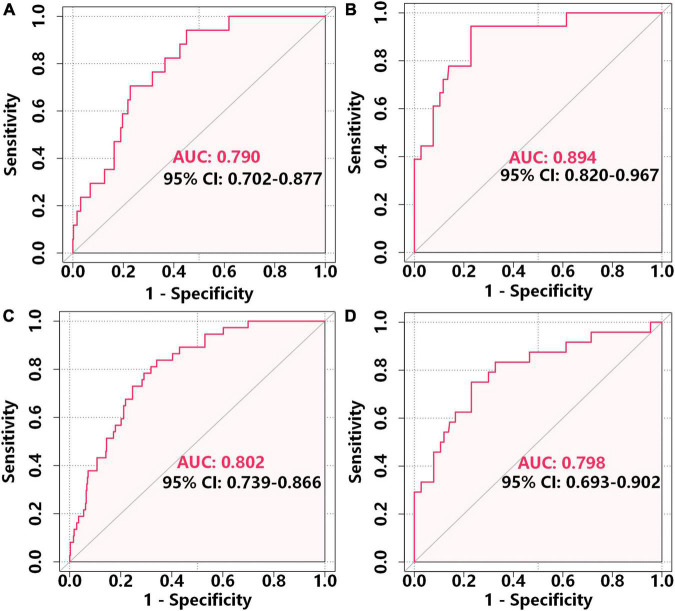
ROC analysis of the accuracy of the nomogram in predicting 1-year MACE-free survival in the development cohort **(A)** and external validation cohort **(B)** and 2-year MACE-free survival in the development cohort **(C)** and external validation cohort **(D)**.

We determined the discriminatory capacity of the model using receiver operating characteristic (ROC) analysis and calibration plots. In the development cohort, ROC analysis indicated the area-under-the-curve (AUC) was 0.790 (95% CI: 0.702–0.877) for prediction of 1-year MACE-free survival and the AUC was 0.802 (95% CI: 0.739–0.866) for prediction of 2-year MACE-free survival, suggesting the model had good predictive performance (Figures 4A,C). Moreover, internal validation using bootstrapping indicated the bias-corrected C-index was 0.750 for 1-year MACE-free survival and 0.774 for 2-year MACE-free survival. Analysis of the independent validation cohort indicated the AUC was 0.894 (95% CI: 0.802–0.967) for prediction of 1-year MACE-free survival and 0.798 (95% CI: 0.693–0.902) for prediction of 2-year MACE-free survival (Figures 4B,D).

**FIGURE 5:**
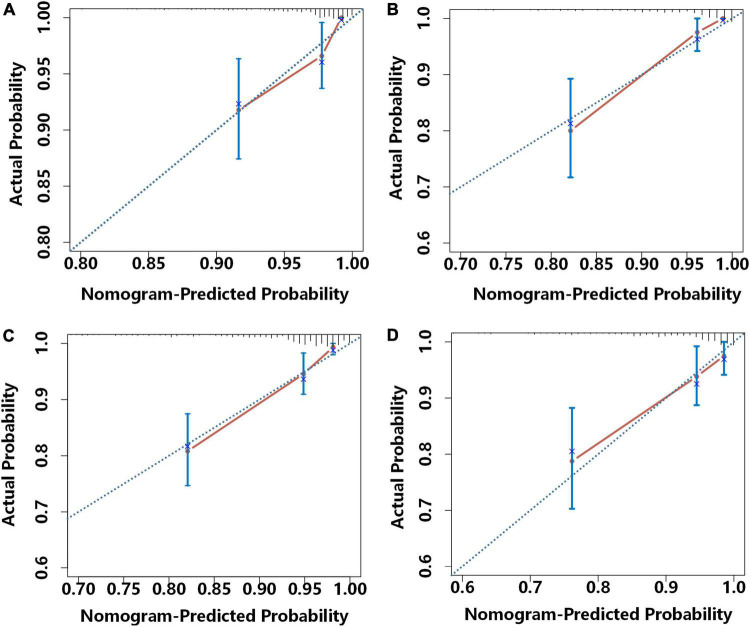
Calibration curves for the prediction of the risk for 1-year MACE-free survival in the development cohort **(A)** and the external validation cohort **(B)** and 2-year MACE-free survival in the development cohort **(C)** and the external validation cohort **(D)**. Each plot shows the relationship of the nomogram-predicted probability (abscissa) and the actual probability (ordinate) Each light blue diagonal line represents the ideal reference line (in which predicted survival probabilities match observed survival rates) and each red line was calculated by bootstrap resampling (500 times), and represents the performance of the nomogram. Thus, a greater similarity of the red and blue lines indicates more accurate predictions of survival.

We further analyzed these data using calibration plots to evaluate the consistency of the actual probabilities with predicted probabilities for 1 and 2-year MACE-free survival in the development cohort (Figures 5A,C) and the external validation cohort (Figures 5B,D). We also used bootstrapping to determine the accuracy of the prediction model. In all cases, there were high correlations between the nomogram predictions and the actual observations.

**FIGURE 6:**
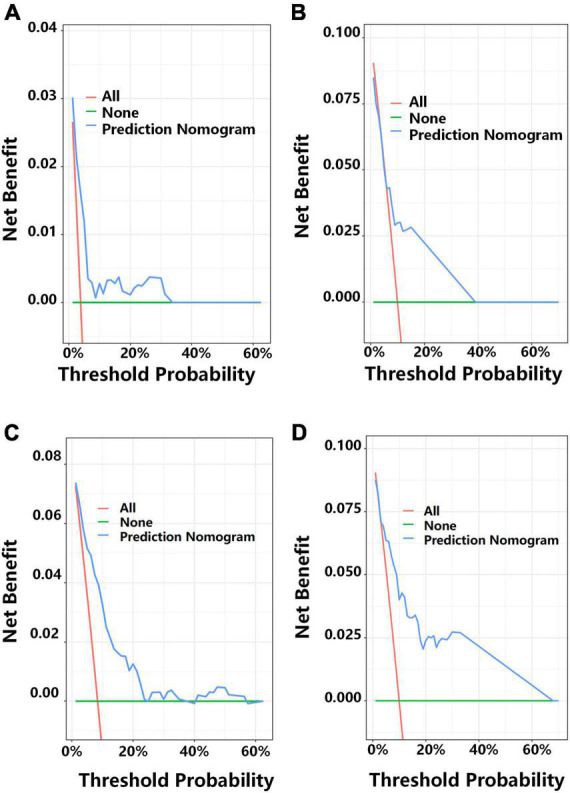
Decision curve analysis (DCA) for predicting 1-year MACE-free survival in the development cohort **(A)** and the external validation cohort **(B)**, and 2-year MACE-free survival in the development cohort **(C)** and the external validation cohort **(D)**. Each plot shows the relationship of threshold probability (abscissa) with the net benefit (ordinate) of the prediction model (blue line), the proportion of patients with MACEs (red line), and the proportion of patients with MACE-free survival (green line).

We then applied DCA to determine the net benefit of the clinical prediction model (Figure 6). In the development cohort, the DCA results indicated that the threshold probability for prediction of MACE-free survival was 2–33% for 1-year MACE-free survival and 2–23% for 2-year MACE-free survival (Figures 6A,C). In the validation cohort, the DCA results indicated that the threshold probability for the prediction model was 5–38% for 1-year MACE-free survival and 5–64% for 2-year MACE-free survival (Figures 6B,D).

**FIGURE 7:**
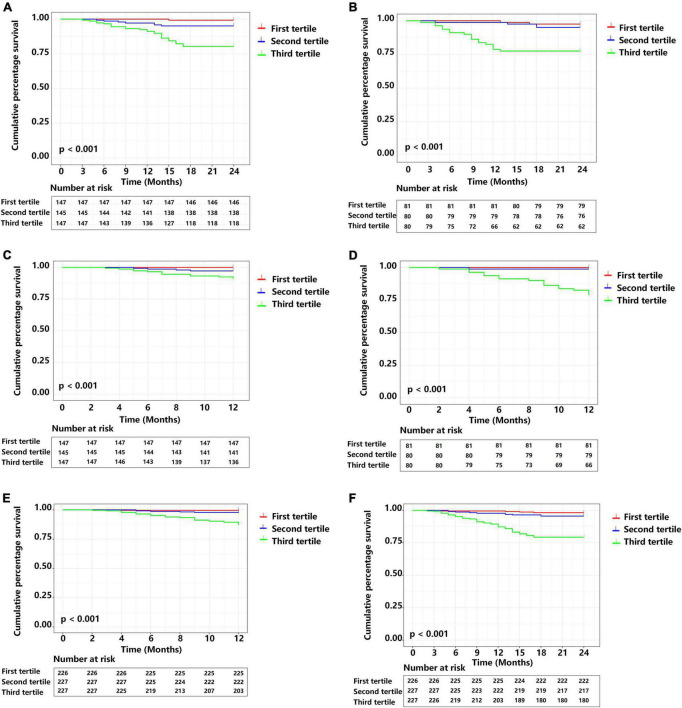
Cumulative MACE-free survival of patients in different prognostic index (PI) tertiles. **(A)** Two year survival in the development cohort. **(B)** Two year survival in the external validation cohort. **(C)** One year survival the development cohort. **(D)** One year survival the external validation cohort. **(E)** One year survival in the complete cohort. **(F)** Two year survival in the complete cohort. Central illustration. Smart wearable electronic devices that use visual and personalized model-based risk assessment and an integrated approach that considers heart rate variability and clinical data (traditional risk factors) could allow patients to receive 24-h real-time personalized home telemonitoring and receive improved clinical management.

We used the total score of each patient in development cohort to stratify patients into a low-risk group (nomogram score < 120.01, *n* = 147), an intermediate-risk group (nomogram score = 120.01–133.72, *n* = 145), and a high-risk group (nomogram score > 133.72, *n* = 147) for analysis of 1 and 2-year MACE-free survival Figures 7A,C). We used the same approach for the validation cohort (Figures 7B,D) and the overall cohort (Figures 7E,F).

Kaplan-Meier analysis of the development cohort (Figures 7A,C) with pairwise comparisons indicated that the risk of MACEs was greater in the high-risk group than the intermediate risk group at 1-year (χ^2^ = 4.898, *P* = 0.027) and 2 years (χ^2^ = 14.630, *P* < 0.001). Similarly, the high-risk group had a higher risk of MACEs than the low risk group at 1 year (χ^2^ = 13.559, *P* < 0.001) and 2 years (χ^2^ = 29.165, *P* < 0.001). Patients in the intermediate-risk group had a higher risk of MACEs than the low risk group at 1 year (χ^2^ = 4.098, *P* = 0.043) and 2 years (χ^2^ = 4.733, *P* = 0.030).

Kaplan-Meier analysis of data from the validation cohorts (Figures 7B,D) also indicated the high-risk group had a greater risk of MACEs than the intermediate-risk group at 1 year (χ^2^ = 15.791, *P* < 0.001) and 2 years (χ^2^ = 10.668, *P* = 0.001). Similarly, patients in the high-risk group had a greater risk of MACEs than the low-risk group at 1 year (χ^2^ = 19.177, *P* < 0.001) and 2 years (χ^2^ = 15.207, *P* < 0.001).

We also performed a Kaplan-Meier analysis of the overall study population (Figures 7E,F). Similar to the results above, patients in the high-risk group had a greater risk of MACEs than the intermediate-risk group at 1-year (χ^2^ = 18.158, *P* < 0.001) and 2-years (χ^2^ = 27.498, *P* < 0.001). Pairwise comparisons also indicated that the high-risk group had greater risk of MACEs than the low-risk group at 1-year (χ^2^ = 27.711, *P* < 0.001) and 2-years (χ^2^ = 40.844, *P* < 0.001).

## Discussion

Our nomograms for predicting the 1 and 2-year probability of MACE-free survival in patients who had ACS used traditional clinical prognostic variables (age, diabetes, smoking, and previous myocardial infarction) and data from non-invasive ambulatory measurements (LF/HF ratio). Thus, we used routinely available clinical variables to develop a nomogram that had reliable discriminative ability, and we validated the nomogram model for predicting the risk of 1 and 2-year MACE-free survival. Our model has potential for use in the risk stratification of patients with ACS who received emergency coronary angiograms. It is possible that inclusion of other predictors in the future may further improve the model.

Acute coronary events may occur in patients who have none of the well-established classical cardiovascular risk factors, suggesting there may be additional coronary risk factors that have not yet been recognized (3–5, 10, 11). Therefore, systematic ACS risk-assessment tools and appropriate recommendations for risk assessment in clinical guidelines are essential for the implementation of strategies that prevent MACEs in these patients. There are several popular models for assessing the risk of cardiovascular disease, but they may not be suitable for use in all populations or countries (14). Moreover, established risk-assessment methods generally consider multiple blood markers, and the need for invasive examination may lead to delays in diagnosis and treatment. For example, the Global Registry of Acute Coronary Events (GRACE) score is widely accepted as a risk stratification tool for the prediction of in-hospital and 6-month all-cause mortality and myocardial infarction (20, 21). Nevertheless, clinicians have not yet optimized implementation of the GRACE score in the risk stratification for death and myocardial infarction at 12-months, particularly in ACS patients who receive standardized treatment (22, 23). Additionally, a retrospective cohort study of 3,982 ACS patients with non-ST-segment elevation found that incorporating available clinical variables, including age, N-terminal pro-brain type natriuretic peptide, and serum creatinine, into a novel ABC score provided better predictions of long-term mortality after PCI than the GRACE score (24). However, the GRACE score and ABC score do not rely on home monitoring of cardiovascular health, a simple, non-invasive, and ambulatory monitoring technique. The use of wearable digital monitoring devices and of individualized risk assessment approaches is increasingly popular. Therefore, daily monitoring of the modulation of cardiac ANS *via* HRV analysis is a valuable, non-invasive, and easy-to-perform method that has relatively good reproducibility, and may provide useful information for the management of patients with ACS.

Our previous study of ACS patients found that automatic evaluation of cardiac ANS function was an effective method for risk-stratified prediction of MACEs when used with GRACE score, in that it provided accurate prognostic information and increased discriminatory ability (22). In addition, for individuals with no prior history of CAD, HRV analysis using remote digital technology may help to improve risk assessment by providing data over a long period of time that can complement classical cardiovascular risk factors. Our previous findings also indicated that HRV correlated with increased adverse cardiovascular events in patients with ACS (12, 25).

Endothelial injury results from the interaction between an imbalanced ANS and inflammation. Failure to maintain lipid homeostasis may result from endothelial injury, and lead to the activation of vascular smooth muscle cells and macrophage infiltration. These pathological events accelerate atherosclerotic plaque formation and cause the deterioration of myocardial ischemia (9, 10, 26, 27). Although an imbalanced ANS is a well-documented risk factor for adverse cardiovascular outcomes (12, 25, 28), few studies have evaluated the use of an integrated approach that incorporates HRV with classical factors for risk stratification. The Singapore Emergency Department Risk Stratification Model (SEDRSM) considers age, gender, heart rate, HRV parameters, and four 12-lead ECG variables to predict 30-day MACEs in patients who present to the emergency department with chest pain (13). However, the SEDRSM was not yet subjected to external validation, and therefore has limited value in clinical practice. Therefore, there is an urgent need for an inexpensive, simple, and non-invasive screening tool for patients with ACS who have a high risk of MACEs, especially for ACS patients who received standardized therapy protocols.

Our results indicated that it is currently simple and feasible to identify the risk of 1 and 2-year MACEs in patients with ACS. We also demonstrated favorable discriminative ability of the nomogram, based on the C-index and AUC values in the development and validation cohorts. Our internal validation (based on bootstrapping) and external validation also indicated the nomogram had satisfactory consistency in the prediction of MACEs. Our study therefore confirmed that specific clinical variables can be used for individualized predictions of risk and risk stratification for MACEs in patients with ACS. Our results may also contribute to the design and implementation of clinical trials that examine individualized clinical decision-making. Importantly, the rapid proliferation of wearable health technologies, such as smart wearable electronic devices that are equipped with green light-emitting diodes for photoplethysmography, for the self-monitoring of health-related parameters, including heart rate, heart-rate variability, and electrocardiograms, are now available in commercially available smartwatches and smartwatch accessories, with arrhythmia notification provided directly to the user (29–31). Thus, future studies could examine the use of smart wearable electronic devices that feature visual and personalized model-based risk assessment based on HRV and traditional risk factors. This may help ACS patients to receive dynamic real-time personalized treatment and management and reduce the high incidence of MACEs in these patients, even when they are at home.

### Study limitations

It is important to note that while our model has significant promise for predicting MACEs in ACS patients who received emergency coronary angiography, there were a few limitations. First, because the sample size was relatively small, the predictive ability of the model needs to be confirmed in large prospective cohort studies. Second, due to the retrospective nature of this study, detailed patient information, such as the GRACE score and the time of ACS onset, was not recorded. Third, although we accounted for several known confounding factors, some unknown confounding factors may have remained that affected HRV. Four, because the two hospitals used different methods for measuring troponin and creatine kinase isoenzyme MB, we did not include these variables in the analysis. Five, because this study was observational, it is unknown if better outcomes could have been achieved from the use of individualized and comprehensive treatment based on other ANS risk models. Six, additional indicators of ANS, such as deceleration capacity, may be also useful as prognostic indicators. Future studies are therefore needed to assess the validity and accuracy of point-of-care testing of cardiac ANS in predicting MACEs after ACS. Finally, for methodological reasons, the ANS can only be assessed using HRV in patients with a sinus rhythm, and the impact of a non-sinus rhythm in patients with ACS remains uncertain.

## Conclusion

Our observational study demonstrated that a risk nomogram that incorporated four conventional clinical characteristics and a single ANS parameter (LF/HF) was a simple method that clinicians can use to assess the risk of MACEs in patients with ACS. We suggest that future large studies are needed for further validation of our nomogram. Nonetheless, the results indicated that our nomogram appeared to be useful for identifying risk of MACEs in patients with ACS, and thus provide a foundation for the use of mobile health monitoring based on alterations of the ANS.

## Data availability statement

Individual participant data that underlie the results reported in this article, after de-identification can be obtained from the corresponding authors upon reasonable request.

## Ethics statement

The studies involving human participants were reviewed, approved and granted for the study by the Local Ethics Committee (Renmin Hospital of Wuhan University; No. WDRY2022-K075; Xiangyang Central Hospital; No.2022-066). The ethics committee waived the requirement of written informed consent for participation.

## Author contributions

HJ, XZ, and HF conceived and performed the experiments. JW and JS performed the experiments and wrote the manuscript. XW and TZ provided expertise and feedback. TX, FY, SD, QD, ZL, FG, XL, YW, and LS provided valuable suggestions and edited the manuscript. All authors contributed to the article and approved the submitted version.
