# Supplementary figures and images for: Prediction of major adverse cardiovascular events in patients with acute coronary syndrome: Development and validation of a non-invasive nomogram model based on autonomic nervous system assessment
Source: Front Cardiovasc Med. 2022 Nov 3;9:1053470. doi: 10.3389/fcvm.2022.1053470 (PMC9670131; doi:10.3389/fcvm.2022.1053470)

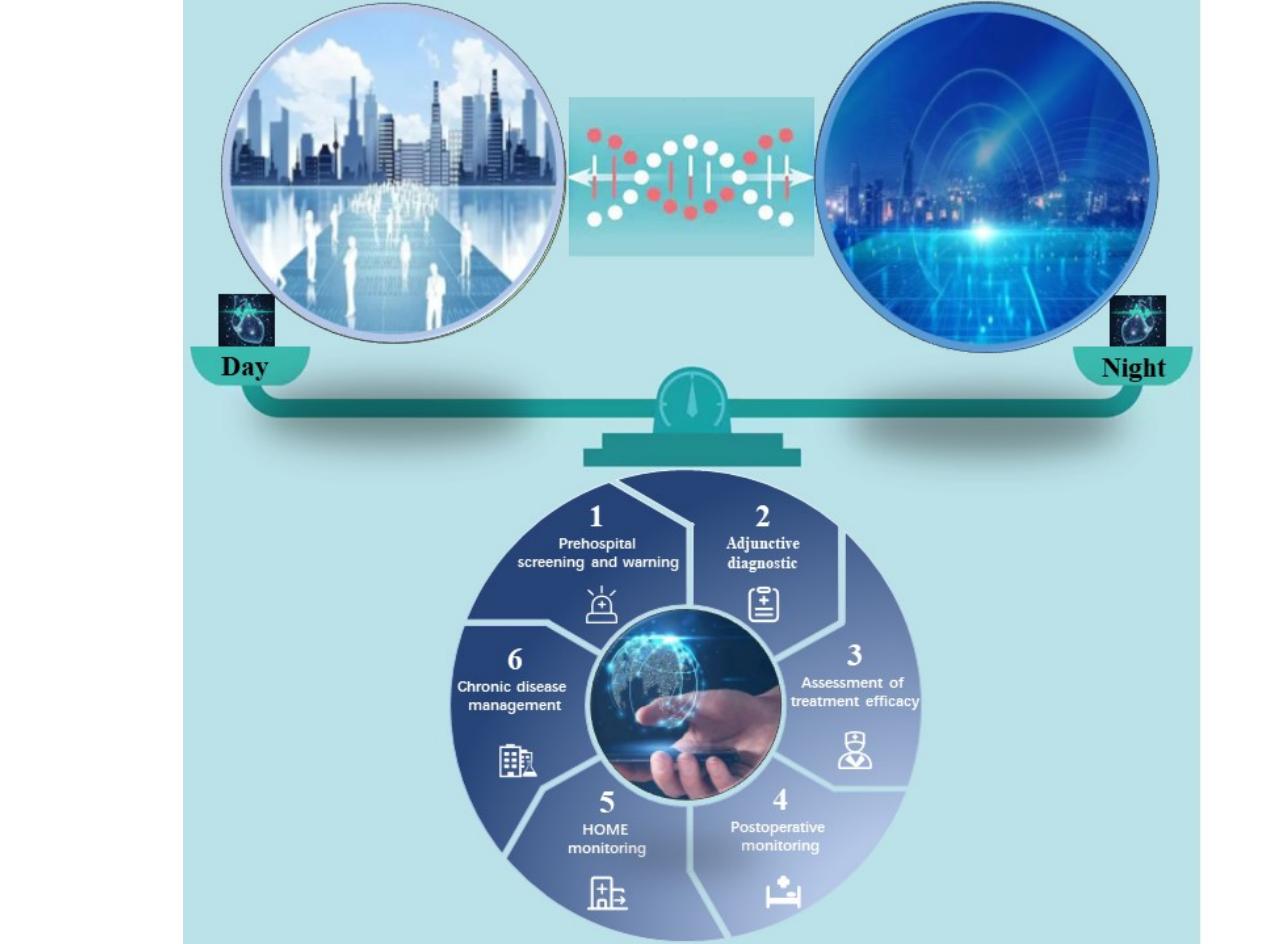

Supplement: Supplementary file 1 [file Image_1.JPEG]
